# Degradome expression profiling in human articular cartilage

**DOI:** 10.1186/ar2741

**Published:** 2009-06-23

**Authors:** Tracey E Swingler, Jasmine G Waters, Rosemary K Davidson, Caroline J Pennington, Xose S Puente, Clare Darrah, Adele Cooper, Simon T Donell, Geoffrey R Guile, Wenjia Wang, Ian M Clark

**Affiliations:** 1 School of Biological Sciences, University of East Anglia, Earlham Road, Norwich NR4 7TJ, UK; 2 Departamento de Bioquímica y Biología Molecular, Facultad de Medicina, Universidad de Oviedo, 33006 Oviedo, Spain; 3 Institute of Orthopaedics, Norfolk & Norwich University Hospital, Colney Lane, Norwich NR4 7UY, UK; 4 School of Computing Sciences, University of East Anglia, Earlham Road, Norwich NR4 7TJ, UK

## Abstract

**Introduction:**

The molecular mechanisms underlying cartilage destruction in osteoarthritis are poorly understood. Proteolysis is a key feature in the turnover and degradation of cartilage extracellular matrix where the focus of research has been on the metzincin family of metalloproteinases. However, there is strong evidence to indicate important roles for other catalytic classes of proteases, with both extracellular and intracellular activities. The aim of this study was to profile the expression of the majority of protease genes in all catalytic classes in normal human cartilage and that from patients with osteoarthritis (OA) using a quantitative method.

**Methods:**

Human cartilage was obtained from femoral heads at joint replacement for either osteoarthritis or following fracture to the neck of femur (NOF). Total RNA was purified, and expression of genes assayed using Taqman^® ^low-density array quantitative RT-PCR.

**Results:**

A total of 538 protease genes were profiled, of which 431 were expressed in cartilage. A total of 179 genes were differentially expressed in OA versus NOF cartilage: eight aspartic proteases, 44 cysteine proteases, 76 metalloproteases, 46 serine proteases and five threonine proteases. Wilcoxon ranking as well as the LogitBoost-NR machine learning approach were used to assign significance to each gene, with the most highly ranked genes broadly similar using each method.

**Conclusions:**

This study is the most complete quantitative analysis of protease gene expression in cartilage to date. The data help give direction to future research on the specific function(s) of individual proteases or protease families in cartilage and may help to refine anti-proteolytic strategies in OA.

## Introduction

Osteoarthritis (OA) is a debilitating degenerative joint disease where degradation of articular cartilage is a key feature [1]. Given the current demographic trend toward an older population, OA – for which age is an important risk factor – will be an increasing health and economic burden on society.

The molecular mechanisms underlying cartilage destruction in OA are poorly understood (see for example [1]). Cartilage is made up of two main extracellular matrix macromolecules: type II collagen and aggrecan, a large aggregating proteoglycan. The former endows the cartilage with its tensile strength, whilst the latter enables cartilage to resist compression. Quantitatively more minor components (for example, type IX, type XI and type VI collagens, biglycan, decorin, cartilage oligomeric matrix protein) also have important roles in controlling the supramolecular organization of the matrix. Normal cartilage extracellular matrix is in a state of dynamic equilibrium, with a balance between synthesis and degradation. For the degradative process there is a balance between proteases that degrade the extracellular matrix and their inhibitors. In OA, the dogma is that a disruption of this balance, in favour of proteolysis, leads to pathological cartilage destruction [2].

Cartilage destruction in OA is thought to be mediated by two main enzyme families; the matrix metalloproteinases (MMPs) are thought to be responsible for cartilage collagen breakdown, whilst enzymes from the ADAMTS (a disintegrin and metalloproteinase domain with thrombospondin motifs) family are thought to mediate cartilage aggrecan loss [3]. Whilst there is strong evidence to support this tenet, there is also evidence that indicates a role for enzymes in other catalytic classes. Examples include serine proteases, which could directly degrade the extracellular matrix or could be involved in potentially rate-limiting activation of proMMPs [4]; similarly, cathepsin K, capable of degrading the collagen triple helix, has also been implicated in cartilage degradation (see for example [5]).

With the completion of the sequencing of several mammalian genomes, the full complement of protease genes has been elucidated [6]. There are 570 human protease genes (not including pseudogenes): 21 aspartate proteases, 154 cysteine proteases, 191 metalloproteases, 176 serine proteases and 28 threonine proteases.

The present study therefore aimed to profile as many of these genes as possible in human cartilage using a quantitative and sensitive RT-PCR approach, and to compare normal tissue with that from patients with OA.

## Materials and methods

### Collection of human cartilage and RNA purification

Human articular cartilage was obtained from femoral heads of patients undergoing total-hip-replacement surgery at the Norfolk and Norwich University Hospital (Norwich, UK). Samples from patients with OA (n = 12, six female patients and six male patients; age range, 37 to 86 years; median age, 72 years; mean age ± standard error of the mean, 68.8 ± 4.2 years) were compared with cartilage from patients undergoing hip replacement following fracture to the neck of femur (NOF) (n = 12, six female patients and six male patients; age range, 68 to 94 years; median age, 84 years; mean age ± standard error of the mean, 81.8 ± 2.4 years). OA was diagnosed using the clinical history and an examination of the patient, coupled with X-ray findings; confirmation of gross pathology was made at the time of joint removal. The fracture patients had no known history of joint disease and their cartilage was free of lesions; 80% of these patients underwent surgery within 36 hours of fracture. This study was performed with Ethical Committee approval, and all patients provided informed consent.

Intact femoral heads were washed in sterile PBS. Cartilage samples were removed from the femoral head using a razor blade, chopped into pieces of 2 to 5 mm, and were snap-frozen in liquid nitrogen within 15 to 30 minutes of surgery. The cartilage was weighed and ground under liquid nitrogen using the Type 6750 Freezer Mill (Spex Certiprep, Glen Creston, Stanmore, UK). RNA was purified essentially following Davidson and colleagues [7]. TRIzol^® ^reagent (Invitrogen Life Technologies, Paisley, UK) was added to ground cartilage (1 ml/0.2 g cartilage), mixed thoroughly and incubated at room temperature for 5 minutes. Ground cartilage was pelleted at 9,500 × *g *for 10 minutes at 4°C, and the supernatant was recovered. Then 300 μl chloroform was added per 0.5 ml TRIzol^®^, vortexed for 15 seconds and incubated at room temperature for 10 minutes. TRIzol^®^/chloroform solution was centrifuged at 9,500 × *g *for 15 minutes at 4°C, and the aqueous layer was recovered into a fresh tube. Then 0.5× volume, 100% ethanol was added and mixed. Using the RNeasy Mini Kit (Qiagen, Crawley, UK), samples were applied to spin columns and centrifuged at 9,500 × *g *for 15 seconds, and the flow-through was discarded. Columns were then washed and eluted according to the manufacturer's instructions. RNA samples were quantified using the NanoDrop^® ^spectrophotometer (NanoDrop Technologies, Wilmington, Delaware, USA) and were stored at -80°C. cDNA was synthesized from 2 μg total RNA using Superscript II reverse transcriptase (Invitrogen) and random hexamers according to the manufacturer's instructions. cDNA was stored at -20°C.

### Quantitative RT-PCR and Taqman^® ^low-density arrays

Quantitative RT-PCR was performed as previously described [7]. Prior to low-density array analysis, samples were assayed for 18S rRNA to ensure that all samples were within 1.5 threshold cycle (Ct) of the median value as a baseline quality control. Samples were also assayed for genes previously shown to be differentially expressed in OA cartilage compared with NOF (*MMP28 *and *ADAMTS16*).

Custom-designed microfluidic Taqman^® ^low-density arrays were obtained from Applied Biosystems (Warrington, UK) with primer sets designed to amplify with similar efficiencies, allowing comparison between genes. The arrays contained 538 protease assays across two microfluidic cards along with 12 housekeeping genes on each card. The Taqman^® ^low-density arrays were used according to the manufacturer's protocol. Briefly, 800 ng cDNA was added to 2× TaqMan^® ^Master Mix (Applied Biosystems) and was loaded onto each card by centrifugation. Relative quantification of genes on the cards was performed using the ABI Prism^® ^7900 HT (Applied Biosystems, Warrington, UK) sequence detection system under the following cycling conditions: 50°C for 2 minutes, 94.5°C for 10 minutes, then 40 cycles of 97°C for 30 seconds, and 59.7°C for 1 minute. The data were analysed using Statminer software (Integromics, Philadelphia, Pennsylvania, USA). The geNorm facility within Statminer identified succinate dehydrogenase subunit A as the most stable housekeeping gene, and the data were therefore normalized to succinate dehydrogenase subunit A expression.

### Statistical analyses

Statistical analysis was by Mann – Whitney U test (either SPSS 16.0, SPSS, Woking, UK, or GraphPad Prism 4, GraphPad Software, La Jolla, USA) or using the LogitBoost-NR algorithm as described below.

#### Machine learning approach for analysing gene expression data

Machine learning methods were applied to the analysis of gene expression data because of their high dimensionality and complexity: in this case, 538 genes and 24 samples.

#### LogitBoost-NR ensemble for classification of samples

A machine learning ensemble can be simply viewed as a combination of a number of models that have been trained independently from the available data of a given problem and then work collectively in order to produce better solutions. The principle behind ensemble learning is that although a classification algorithm may only be able to produce a model with slightly better accuracy than random guessing, if several such models are produced and combined into an ensemble, their combined accuracy will be greater than any single classifier, providing they are sufficiently diverse from each other to avoid making similar errors, and boosting algorithms are designed with the aim of producing a high level of diversity.

Ensemble classification methods such as boosting have been applied to the classification of gene expression data and have produced more accurate results [8] than the individual models that work alone. Boosting algorithms such as LogitBoost [9] iteratively employ another classification algorithm known as the base learner to learn from the data samples and generate a series of models. In the case of gene expression data, the most common base learner used is the decision tree or decision stump, which is a decision tree consisting of a single node. Initially all samples are assigned equal weights for training the first model or classifier. Then the accuracy of the produced model is measured and the weights of individual samples are adjusted so that the weights of misclassified samples are increased (that is, boosted) while those of correctly classified samples are reduced. At the next iteration the base learner will concentrate on learning the information represented by the misclassified samples. This boosting process goes on until a preset stopping criterion (such as either all of the samples have been learned correctly or a fixed number of iterations) is met. After the boosting process a series of models is therefore produced with the sample weights being possibly adjusted at each iteration. These models are then combined to form an ensemble of classifiers. The ensemble is then validated and tested using different data samples before being used for classifying new samples by combining the outputs of the models by simple majority or weighted voting.

The LogitBoost-NR algorithm [10] is an extension of LogitBoost [9] and was specifically designed for the classification of gene expression data. This was achieved by incorporating feature nonreplacement, where the data features (genes) used to construct a model at a given round of boosting are not available at subsequent rounds. This ensures that the models constructed at different boosting rounds use different genes, which helps to achieve a high diversity between the models in the ensemble. Such an approach is particularly appropriate in conditions such as OA where many genes may be significant to the pathology of the disease. Boosting algorithms are also able to produce accurate predictive ensembles when the number of features (genes) in the data is much larger than the number of samples, as is the usually the case with gene expression data, whereas conventional techniques such as logistic regression are unable to do this. More details of the LogitBoost-NR methodology can be found in Additional data file 1.

#### Boosting ensemble for gene selection/ranking

The particular genes used in a classification ensemble produced by boosting as described above can be reasonably assumed to be the most important in the pathology of the disease in question, and a method for ranking genes based on LogitBoost-NR is described in Guile and Wang [10]. In this method a training dataset consisting of the data for two-thirds of the samples is randomly partitioned from a complete dataset. The LogitBoost-NR algorithm with decision stumps as base learner is then applied for 25 iterations of boosting to construct a classification ensemble using 25 different genes. The process is repeated for 50 different random partitions of the data and the genes are scored according to the frequency of their presence in the ensembles generated. A gene used in all 50 ensembles therefore receives a score of 50, while a gene that is only used once receives a score of 1. The genes are then ranked according to their scores.

This ranking method was found to be much more effective than the Wilcoxon test for selecting the genes most useful for predictive classification of DNA microarray data [10]. We applied this method to the gene expression data obtained for the present study to obtain a ranking of the genes. We tested this by performing predictive classification using the top-ranked genes with LogitBoost-NR. Because of the small number of samples available in the present study compared with the microarray datasets originally used for developing the LogitBoost-NR classification and gene selection methods [8,10], we used equal-sized training and testing datasets of the data when making the train:test splits, rather than two-thirds:one-third.

## Results

At the time of assay design, the Taqman^® ^low-density array format allowed us to assay 538 of the 570 human proteases (94%; 21 aspartic proteases, 139 cysteine proteases, 188 metalloproteases, 162 serine proteases and 28 threonine proteases). At the time of writing there are an additional 15 assays available in this format, so 553 genes could now be assayed (97%). These 538 assays were split across two Taqman^® ^low-density array cards along with a number of controls (for example, replicates, housekeeping genes, extracellular matrix genes).

We used the median Ct of each gene (without normalization) as an approximate measure of its expression, assigning 20 < Ct < 25 as very high, 25 < Ct < 30 as high, 30 < Ct < 35 as moderate, 35 < Ct < 40 as low, and Ct = 40 as not detected, based on our previous experience [11]. Table 1 presents the spread of gene expression in each of these bands and demonstrates that the majority of genes are expressed at the moderate to high level. Moreover, there is a general increase in gene expression in the OA samples, with the largest change being within the metalloproteases where several genes move from the not detected or low bands up to the moderate band.

**Table 1:** Expression level of protease genes in each catalytic class

Expression level	Aspartate proteases		Cysteine proteases		Metalloproteases		Serine proteases		Threonine proteases	
					
	NOF	OA	NOF	OA	NOF	OA	NOF	OA	NOF	OA
20 < Ct < 25	0	1	2	4	2	4	3	3	0	0
25 < Ct < 30	10	10	85	80	70	74	47	51	16	17
30 < Ct < 35	4	5	33	38	52	70	29	33	8	6
35 < Ct < 40	2	1	8	6	17	5	19	21	1	2
ND	5	4	11	11	47	35	64	54	3	3

NOF, fracture to the neck of femur; OA, osteoarthritis; Ct, threshold cycle; ND, not detected (median Ct = 40).

We included 12 frequently used housekeeping genes on each Taqman^® ^low-density array and used the geNorm algorithm to select the most stable gene for normalization of the data [12]. Hence, all data were normalized to expression of succinate dehydrogenase subunit A (a gene encoding a protein constituent of the mitochondrial respiratory chain).

**Figure 1:**
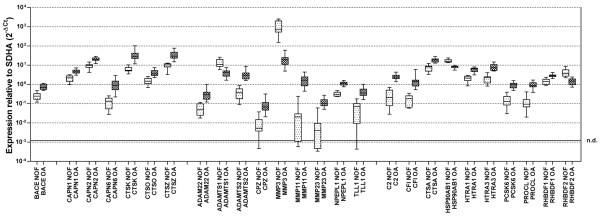
Genes showing most significant differential expression between osteoarthritis and fracture groups. Box and whisker plot for all genes displaying a statistical significance of *P *< 0.0001 by Mann-Whitney U test. For full gene names, see Tables 3 to 6. SDHA, succinate dehydrogenase subunit A; n.d., not detected.

Table 2 presents the numbers of genes in each catalytic class that are differentially expressed in OA and NOF with a significance of *P *< 0.05 in a pairwise (OA vs. NOF) Mann – Whitney U test. Figure 1 shows a box and whisker plot for the 26 genes showing *P *< 0.0001 between these two groups.

**Table 2:** Number of genes in each catalytic class showing differential expression between osteoarthritis and fracture groups

	*P *< 0.05	*P *< 0.01	*P *< 0.001	*P *< 0.0001
Aspartic proteases	1	3	3	1
Cysteine proteases	17	15	6	6
Metalloproteases	19	25	23	9
Serine proteases	19	11	6	10
Threonine proteases	3	2	0	0

Numbers of genes showing statistical significance between the two groups (osteoarthritis versus fracture to the neck of femur (NOF)) in each catalytic class. Pairwise statistical analysis between groups was performed using the Mann-Whitney U test.

Tables 3, 4, 5 and 6 show the fold change between the median values of OA and NOF for all of the genes in each catalytic class that are differentially expressed with a statistical significance of *P *< 0.01 in the pairwise analysis. The median Ct is included as an indication of expression level (as discussed above). Eight genes (*IHH*, *ADAM28*, *ADAM33*, *ASPA*, *CRMP1*, *MMP15*, *MMP28 *and *PCSK2*) are not expressed in NOF (that is, median Ct = 40) but are expressed (35 > Ct > 32) in OA cartilage. Details of all genes analysed can be found in Additional data file 2.

**Table 3:** Fold change and threshold cycle for aspartate proteases showing significant difference between OA and NOF

Gene name	Gene symbol	*P *value	Fold change OA/NOF	Median threshold cycle
β-Secretase 1	*BACE1*	0.00005	2.9	30.7
Nuclear receptor interacting protein 2	*NRIP2*	0.00014	3.7	33.8
Presenilin homolog 4/SPPL2B	*SPPL2B*	0.00028	1.9	28.1
Cathepsin D	*CTSD*	0.00071	3.4	24.5
Presenilin 2	*PSEN2*	0.00137	1.9	30.1
Nuclear receptor interacting protein 3	*NRIP3*	0.00170	3.7	34.6
Pepsin A	*PGA3/4/5*	0.00170	6.8	35.3

Fold change and median threshold cycle for all aspartate protease genes showing significant difference at *P *< 0.01 between osteoarthritis (OA) versus fracture to the neck of femur (NOF). *P *value from the Mann-Whitney U test.

**Table 4:** Fold change and threshold cycle for cysteine proteases showing significant difference between OA and NOF

Gene name	Gene symbol	*P *value	Fold change OA/NOF	Median threshold cycle
Cathepsin K	*CTSK*	0.00005	5.2	26.0
Cathepsin Z	*CTSZ*	0.00005	2.9	25.4
Calpain 2	*CAPN2*	0.00006	2.0	25.5
Calpain 1	*CAPN1*	0.00008	2.0	27.5
Calpain 6	*CAPN6*	0.00008	6.8	30.9
Cathepsin O	*CTSO*	0.00008	2.4	28.2
Autophagin-2	*AUTL2*	0.00014	2.0	30.3
Cathepsin H	*CTSH*	0.00022	3.9	30.1
γ-Glutamyl hydrolase	*GGH*	0.00028	2.4	31.6
Cathepsin C	*CTSC*	0.00071	3.4	28.7
Bleomycin hydrolase	*BLMH*	0.00089	1.5	30.1
Ubiquitin-specific protease 19	*USP19*	0.00089	1.5	27.8
Ubiquitin-specific protease 18	*USP18*	0.00137	2.4	32.4
Cathepsin L	*CTSL*	0.00170	0.40	24.8
Ubiquitin-specific protease 13	*USP13*	0.00256	0.61	28.2
Calpain 5	*CAPN5*	0.00464	2.3	31.4
Ubiquitin-specific protease 37	*USP37*	0.00464	0.46	30.1
Caspase-8	*CASP8*	0.00561	1.5	30.0
Cathepsin F	*CTSF*	0.00561	1.8	25.7
Ubiquitin-specific protease 36	*USP36*	0.00561	0.56	29.3
Cathepsin W	*CTSW*	0.00677	2.1	34.6
Ubiquitin C-terminal hydrolase 3	*UCHL3*	0.00677	1.2	30.3
Ubiquitin-specific protease 28	*USP28*	0.00813	0.58	29.1
Caspase-2	*CASP2*	0.00974	1.2	28.6
hGPI8	*PIGK*	0.00974	1.9	27.8
Sentrin/SUMO protease 2	*SENP2*	0.00974	0.56	27.4

Fold change and median threshold cycle for all cysteine protease genes showing significant difference at *P *< 0.01 between osteoarthritis (OA) versus fracture to the neck of femur (NOF). *P *value from the Mann-Whitney U test. SUMO, small ubiquitin-like modifier.

**Table 5:** Fold change and threshold cycle for metalloproteases showing significant difference between OA and NOF

Gene name	Gene symbol	*P *value	Fold change OA/NOF	Median threshold cycle
ADAM22	*ADAM22*	0.00005	6.0	32.3
ADAMTS2	*ADAMTS2*	0.00005	8.1	29.1
Aminopeptidase-like 1	*NPEPL1*	0.00005	3.7	30.2
Stromelysin 1	*MMP3*	0.00005	0.03	22.5
Stromelysin 3	*MMP11*	0.00005	81.4	31.0
MMP23A/B	*MMP23A/B*	0.00006	24.7	34.2
Mammalian tolloid-like 1 protein	*TLL1*	0.00006	4.9	31.9
ADAMTS1	*ADAMTS1*	0.00008	0.37	26.3
Carboxypeptidase Z	*CPZ*	0.00008	12.6	35.0
Procollagen C-proteinase	*BMP1*	0.00011	2.9	29.2
MT3-MMP	*MMP16*	0.00014	6.9	31.2
ADAMTS14	*ADAMTS14*	0.00017	11.2	33.5
ADAMTS16	*ADAMTS16*	0.00017	19.6	33.7
Aminopeptidase N	*ANPEP*	0.00017	5.3	27.2
Dihydropyrimidinase-related protein 2	*DPYSL2*	0.00022	2.2	25.1
Pappalysin-2	*PAPPA2*	0.00022	0.28	30.4
Plasma Glu-carboxypeptidase	*PGCP*	0.00022	2.9	29.9
Cytosol alanyl aminopeptidase	*NPEPPS*	0.00028	0.69	25.7
Gelatinase A	*MMP2*	0.00036	9.7	24.4
Leucyl aminopeptidase	*LAP3*	0.00045	1.6	28.9
ADAMTS9	*ADAMTS9*	0.00057	0.10	30.4
Gelatinase B	*MMP9*	0.00057	31.9	31.3
Aminopeptidase B-like 1	*RNPEPL1*	0.00071	2.1	29.5
Membrane dipeptidase 2	*DPEP2*	0.00071	5.6	35.1
NAALADASE II	*NAALAD2*	0.00071	3.0	35.0
PM20D2 peptidase	*PM20D2*	0.00071	0.36	27.6
ADAM12	*ADAM12*	0.00089	4.0	27.6
MMP19	*MMP19*	0.00089	16.5	29.8
MMP21	*MMP21*	0.00089	3.2	34.1
Carboxypeptidase X1	*CPXM1*	0.00137	36.1	31.6
ADAMTS12	*ADAMTS12*	0.00170	20.3	32.3
Adipocyte-enhanced binding protein 1	*AEBP1*	0.00170	2.2	24.6
Collagenase 3	*MMP13*	0.00170	26.0	27.1
ADAM9	*ADAM9*	0.00256	1.8	27.3
FACE-2/RCE1	*FACE2*	0.00256	0.80	29.6
X-Pro dipeptidase	*PEPD*	0.00256	2.1	31.2
Leukotriene A4 hydrolase	*LTA4H*	0.00313	0.51	26.7
NAALADASE like 2	*NAALADL2*	0.00313	3.8	31.0
Neprilysin	*MME*	0.00313	9.8	30.9
ADAM8	*ADAM8*	0.00464	2.0	34.1
Aminoacylase	*ACY1*	0.00464	1.6	33.2
O-Sialoglycoprotein endopeptide	*OSGEP*	0.00464	1.8	29.7
ADAMTS7	*ADAMTS7*	0.00561	15.0	34.0
Dihydroorotase	*CAD*	0.00561	1.4	28.1
ADAMTS6	*ADAMTS6*	0.00677	2.0	31.3
FACE-1/ZMPSTE24	*FACE1*	0.00677	1.8	30.9
Glutaminyl cyclase 2	*QPCTL*	0.00677	0.65	29.4
OMA1	*OMA1*	0.00813	0.50	27.6
Archaemetzincin-1	*AMZ1*	0.00974	0.58	31.4
Collagenase 1	*MMP1*	0.00974	0.24	30.8
Neurolysin	*NLN*	0.00974	1.6	30.7

Fold change and median threshold cycle for all metalloprotease genes showing significant difference at *P *< 0.01 between osteoarthritis (OA) versus fracture to the neck of femur (NOF). *P *value from the Mann-Whitney U test. ADAM, a disintegrin and metalloproteinase domain; ADAMTS, a disintegrin and metalloproteinase domain with thrombospondin motifs; MMP, matrix metalloproteinase.

**Table 6:** Fold change and threshold cycle for serine and threonine proteases significant between OA and NOF

Gene name	Gene symbol	*P *value	Fold change OA/NOF	Median threshold cycle
Complement component 2	*C2*	0.00005	11.7	29.6
Complement factor I	*CFI*	0.00005	7.8	30.5
Heat shock 90 kDa protein 1 beta	*HSP90AB1*	0.00005	0.54	25.4
HTRA3	*HTRA3*	0.00005	3.3	27.3
Lysosomal carboxypeptidase A	*CTSA*	0.00005	2.3	25.8
Osteoblast serine protease	*HTRA1*	0.00005	3.0	27.7
PACE4 proprotein convertase	*PCSK6*	0.00005	7.5	30.8
Protein C-like	*PROCL*	0.00006	9.3	30.6
Rhomboid 5 homolog 1	*RHBDF1*	0.00006	2.0	28.3
Rhomboid 5 homolog 2	*RHBDF2*	0.00008	0.40	27.9
Complement factor B	*CFB*	0.00014	0.43	27.5
Seprase	*FAP*	0.00017	4.0	28.0
Proprotein convertase 7	*PCSK7*	0.00022	1.8	28.6
Epoxide hydrolase	*EPHX1*	0.00057	2.1	30.1
Presenilins-associated rhomboid like	*PARL*	0.00071	0.69	26.8
Rhomboid domain containing 1	*RHBDD1*	0.00071	0.58	26.1
Glycosylasparaginase	*AGA*	0.00111	1.9	29.9
Heat shock protein 90 kDa beta	*HSP90B1*	0.00137	0.63	24.5
HTRA4	*HTRA4*	0.00137	3.6	32.1
Serine carboxypeptidase 1	*SCPEP1*	0.00209	1.9	26.3
Complement factor D	*DF*	0.00313	3.8	31.0
Kallikrein hK4	*KLK4*	0.00382	10.3	34.4
Vitellogenic carboxypeptidase-L	*CPVL*	0.00382	6.2	31.6
Proprotein convertase 1	*PCSK1*	0.00464	0.10	30.7
Reelin	*RELN*	0.00464	5.6	30.0
Dipeptidyl-peptidase II	*DPP7*	0.00813	1.6	26.1
Lysosomal Pro-X C-peptidase	*PRCP*	0.00974	1.7	27.2
Proteasome catalytic subunit 1	*PSMB6*	0.00974	1.5	27.2

Fold change and median threshold cycle for all serine and threonine protease genes showing significant difference at *P *< 0.01 between osteoarthritis (OA) versus fracture to the neck of femur (NOF). *P *value from the Mann-Whitney U test.

**Figure 2:**
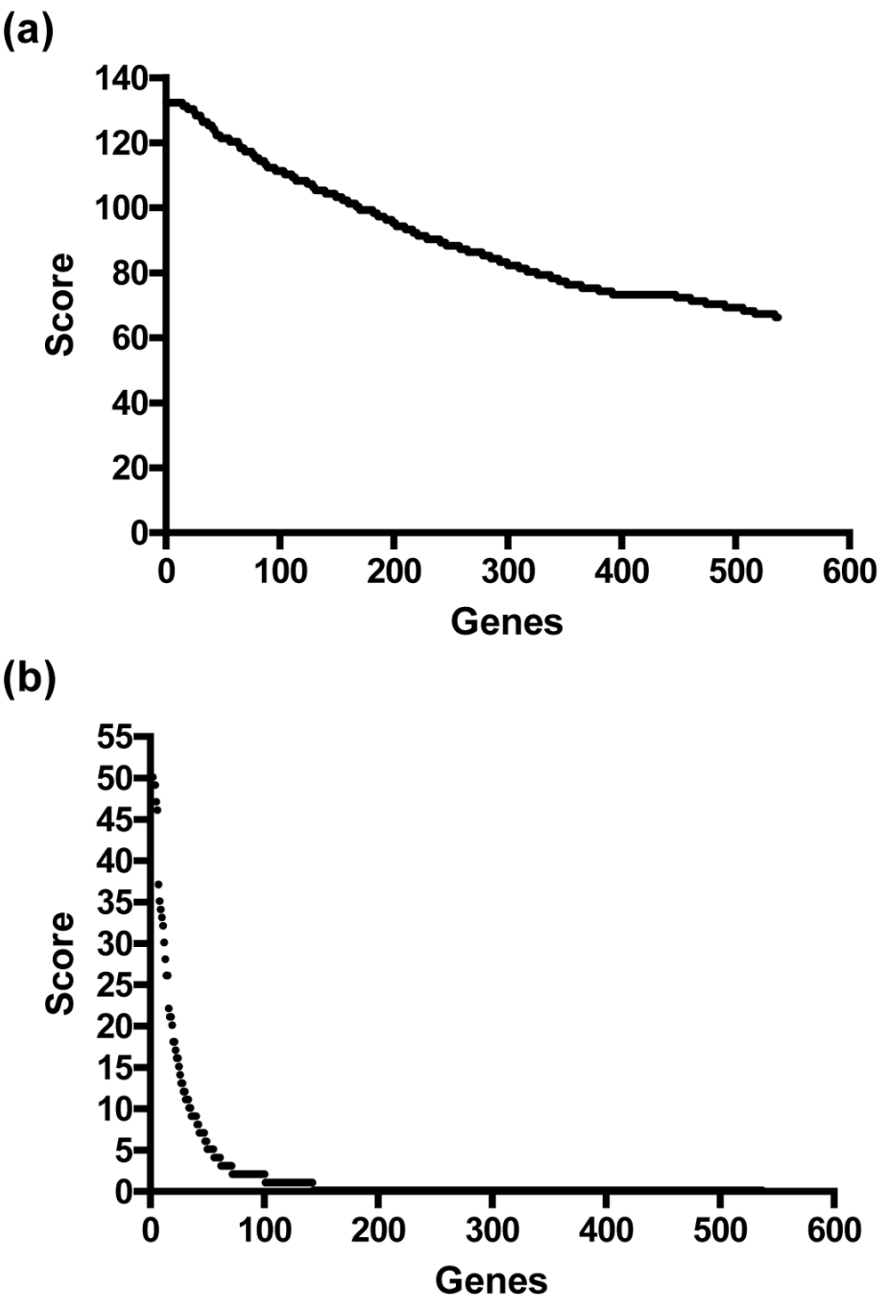
Ranking of genes. **(a) **Rank scores by the Wilcoxon text. **(b) **Rank scores by the LogitBoost-NR algorithm.

The simple analyses above demonstrate that assigning relative importance to any gene in distinguishing OA from NOF is not trivial. Unsupervised cluster analysis shows that the samples are separated into OA and NOF based on their gene expression profiles (data not shown). Ranking the genes using the Wilcoxon test yielded a relative order of importance in the ability of each gene to distinguish OA from NOF, but the rank scores decrease relatively slowly across the genes such that all genes are assigned at least some importance and the difference between the most and least important is small (Figure 2a). Similarly the top 15 genes are given identical rank and are thus impossible to separate further. We therefore employed a more sophisticated machine learning method originally developed for DNA microarray data analysis, based on the ensemble learning algorithm LogitBoost-NR as described in [10], to provide a second ranking of the genes. Figure 2b shows the ranking scores, demonstrating an enhanced ability to assign relative importance to each gene and also to exclude genes with no contribution to separating the two groups, compared with the standard methodology.

Table 7 presents the top 30 genes ranked by the LogitBoost-NR algorithm with the Wilcoxon score for comparison. There is broad similarity across the top genes ranked by both methods, although there is divergence – for example, heat shock 90 kDa protein 1 beta (*HSP90AB1*) is ranked equal top by Wilcoxon but below the top 100 by LogitBoost-NR, and therefore is not presented in the table. Full details of each ranking can be found in Additional data file 3.

**Table 7:** Ranking of genes using the LogitBoost-NR algorithm compared with the Wilcoxon test

Gene symbol	Rank by LogitBoost-NR	Rank by Wilcoxon
*BACE1*	50	132
*CTSK*	50	132
*CTSZ*	50	132
*MMP3*	49	132
*MMP11*	49	132
*ADAM22*	47	132
*ADAMTS2*	46	132
*CAPN2*	37	131
*MMP23A*	35	131
*ADAMTS1*	34	130
*TLL1*	33	131
*NPEPL1*	32	132
*CTSO*	30	130
*CAPN6*	28	130
*CAPN1*	26	130
*NPEPPS*	26	125
*CTSH*	22	126
*BMP1*	21	129
*NRIP2*	21	128
*MMP16*	20	128
*C2*	18	132
*AUTL2*	18	128
*CTSD*	17	121
*CFI*	16	132
*PROCL*	16	131
*CPZ*	15	130
*USP19*	14	120
*MMP2*	13	124
*SPPL2B*	13	122
*HTRA1*	12	132

For the top 30 genes ranked by LogitBoost-NR. For full gene names, see Tables 3 to 6.

## Discussion

The investigation of proteolysis in cartilage has been confined to subsets of each catalytic class. Extracellular proteolysis particularly has been focused on the matrix-degrading metzincins from the MMP and ADAMTS families. The aim of the present study was to gain quantitative expression data for the majority of proteases, both intracellular and extracellular, in all catalytic classes.

Validation of the Taqman^® ^low-density array data in this study can be achieved in part by comparison with our previously published data for the MMP and ADAMTS families in similar tissue cohorts. Of the 27 genes in these families shown to be differentially expressed in NOF compared with OA in the present study, 25 genes were regulated similarly in an earlier cohort [7]. For *MMP14 *(*P *= 0.03) and *ADAMTS6 *(*P *= 0.007), we had not previously reported significant differences between NOF and OA. Similarly, six genes shown to be differentially expressed in the earlier cohort [7] were not identified as such in the current study. Of these, *MMP8*, *MMP10*, *ADAMTS3 *and *ADAMTS10 *all exhibited the same increase or decrease in expression between the NOF and OA, but this did not reach statistical significance. *MMP12 *and *ADAMTS20 *were not detected in the current study and were detected only at low levels in the earlier cohort [7]. These differences probably reflect variation between cohorts, variation in assay methodology or primer sets used, inaccuracy in the assay itself where expression levels are very low and/or problems of multiple testing. No correction for multiple testing has been applied in our analyses of the data since this can often lead to a type two error (false negatives). This would limit the utility of gene expression studies where the validity of any multiple testing procedure has yet to be ascertained [13].

We used two methods to assign significance to the genes assayed, a standard Wilcoxon/Mann – Whitney U-test method and the LogitBoost-NR methodology. The genes identified as being most significant by the two different methods were broadly similar, increasing confidence that these genes are the most important. Whilst there are too many proteases to review the potential role of each in OA individually, it is worth providing details of the most significantly regulated genes.

*BACE1*, the aspartic protease β-secretase, catalyses the rate-limiting step in the production of amyloid beta, leading to plaque formation in Alzheimer's disease [14]. A number of substrates other than amyloid precursor protein have been described for *BACE1*, although the focus has been on the central nervous system. These include the shedding of the ectodomain of IL-1 receptor type II – a decoy receptor that acts as a ligand sink – from the cell surface, thereby limiting the action of IL-1 [15]. Interestingly, in chondrocytes, insulin-like growth factor (IGF)-1 has been shown to induce the level of IL-1 receptor type II as a mechanism to counter the catabolic effects of IL-1 [16]. An increase in *BACE1 *activity could therefore potentiate IL-1 signalling, contributing to cartilage destruction in OA.

A major class of ectodomain sheddases is the ADAM (a disintegrin and metalloproteinase domain) family. *ADAM12*, reported additionally to cleave some matrix components as well as IGF binding proteins, has been linked to OA in genetic association studies (for example [17]). *ADAM8 *is expressed in the developing skeleton [18] and has been shown, along with *ADAM23*, to be expressed during differentiation of mesenchymal stem cells into chondrocytes [19]. The most significant difference in ADAM expression in OA compared with normal is for *ADAM22*. This protein has no protease activity and is presumed to have roles in cell adhesion or as a receptor (with several binding proteins identified), particularly in the nervous system since the *ADAM22 *null mouse displays ataxia and peripheral nerve hypomyelination [20].

Following ectodomain shedding, some transmembrane proteins undergo so-called regulated intramembrane proteolysis [21], whereby the peptide bond is cleaved within the hydrophobic lipid bilayer, often releasing the cytoplasmic domain for intracellular action. The best known of these intramembrane cleaving proteases is γ-secretase, which cleaves amyloid precursor protein in the second step of amyloidogenesis. One component of this enzyme is presenilin 2, also significantly increased in expression in OA cartilage in this study. γ-Secretase has recently been shown to process the IL-1 receptor type I, the signalling receptor [22]. Other intramembrane cleaving proteases come from the S2P (MBTPS1 and MBTPS2), the signal peptide peptidases (or presenilin homologues) and the rhomboids [23]. Many genes across these families show significant changes in expression between normal and OA cartilage, and the potential to act in inflammatory pathways – for example, *SPPL2B *(presenilin homolog 4) has been shown to promote intramembrane proteolysis of TNFα [24].

Two aspartic proteases significantly increased in expression in OA are nuclear hormone interacting proteins (*NRIP2 *and *NRIP3*). These proteases are recently discovered and have not been reported in cartilage before, but nuclear hormone receptors have many roles in cartilage homeostasis.

Cathepsin D is a lysosomal enzyme, capable of aggrecan cleavage. Whilst a recent proteomics study confirms that cathepsin D is highly expressed in chondrocytes [25], its role in cartilage degradation remains equivocal.

Of the cysteine protease cathepsins, cathepsin K showed a high level of expression in cartilage, as well as a robust and significant increase in expression in OA. Cathepsin K is the only vertebrate enzyme outside the MMP family capable of degrading the collagen triple helix, but has also been shown to degrade other matrix proteins. Cathepsin K activity has been demonstrated in human articular cartilage and has been shown to play a role in collagen cleavage in at least a subset of OA patients [5].

Cathepsin O and cathepsin Z are also highly expressed genes in cartilage and, again, significantly increased in expression in OA tissue versus normal tissue. Little is known about the function of each of these enzymes, although they are presumed to be active predominantly intracellularly [26,27]. Cathepsin H, cathepsin C, cathepsin F, cathepsin W, cathepsin B and cathepsin S are also increased in expression in OA tissue compared with normal tissue, with the converse being true for cathepsin L.

The two classical calpains, calpain I (μ-calpain, the catalytic subunit encoded by *CAPN1*) and cathepsin II (m-calpain, the catalytic subunit encoded by *CAPN2*), are both highly expressed in articular cartilage and significantly increased in OA. This is also true for the related nonclassical calpains encoded by *CAPN5 *and *CAPN6*. At least calpain II is capable of cleaving aggrecan, with evidence for cleavage at the calpain-sensitive site [28]. The expression of both calpain I and cathepsin II can be induced by TNFα, whilst μ-calpain may regulate TNFα induction of *MMP3*, at least in rheumatoid synovial cells [29].

Hypertrophic chondrocytes in the growth plate undergo programmed cell death, with recent evidence pointing to the process of autophagy rather than (or as well as) classical apoptosis. The same processes may occur during OA where programmed cell death is also thought to occur [30]. Many proteases are involved in these processes, caspases are involved in classical apoptosis, autophagins are involved in the formation of the autophagosome in autophagy, and lysosomal cathepsins are involved in the degradation of proteins within lysosomes [31]. Several of these proteases show regulation in OA cartilage in the current study.

Related to these observations, mice deficient in the metalloprotease Zmpste24 display a progeria syndrome similar to human Hutchinson – Gilford progeria and a concomitant increase in autophagy [32]. One Zmpste null line displays some growth plate phenotype [33]. Zmpste24 and the related protease Rce1 are both altered in expression in OA cartilage, although the increase in Zmpste24 is difficult to explain in terms of these observations.

There are also many proteases involved in the removal of ubiquitin (and ubiqutin-like) modifications from proteins, thereby impacting upon protein degradation, intracellular localization and epigenetic modification. These include the ubiquitin C-terminal hydrolases, ubiquitin-specific proteases, OTU-domain-containing proteases and SUMO (small ubiquitin-like modifier) proteases, many of which are expressed in cartilage and regulated during OA in the present study. This obviously has the potential to impact upon many areas of cell function – including, for example, transforming growth factor beta signalling, where the action of Smurf2, an E3 ubiquitin ligase, may lead to degradation of Smad proteins, reduced transforming growth factor beta signalling and OA-like changes [34]. Sox9, the key transcription factor in chondrogenesis, is also subject to ubiquitination and proteosomal degradation, regulating transcriptional activity [35]. Interestingly, the expression of ubiquitin itself is significantly decreased in OA cartilage compared with normal in our sample cohort (data not shown).

γ-Glutamyl hydrolase is another cysteine protease showing a significantly increased expression in OA cartilage. This enzyme is involved in folate metabolism, which has been reported as necessary to chondrocytes for correct growth and differentiation [36].

The metzincins (MMPs, ADAMs and ADAMTSs) have been discussed above and previously [7], but the expression of several other metalloproteases is altered in OA cartilage. Along with *ADAMTS2 *and *ADAMTS14 *(collagen N-propeptidases), both tolloid-like 1 and *BMP1 *(collagen C-propeptidases) are also increased in expression in OA. The same is true for the *COL2A1 *gene and indeed *COL1A1 *and *COL1A2 *genes in our samples (data not shown), reflecting an increased collagen synthesis previously described in OA cartilage [1].

Carboxypeptidase Z removes carboxyl-terminal basic amino acids from proteins and has been shown to modulate Wnt signalling in the developing skeleton, with the cysteine-rich domain acting as a binding site for Wnts. In the growth plate, carboxypeptidase Z is co-expressed with Wnt4, although this is not true in our articular cartilage samples (data not shown). Overexpression of carboxypeptidase Z activates Wnt signalling and promotes the terminal differentiation of growth plate chondrocytes [37].

Four aminopeptidases or aminopeptidase-like enzymes are amongst the metalloprotease genes most significantly increased in expression in OA cartilage. Aminopeptidase N is identical to CD13, a cell surface marker used to identify mesenchymal stem cells [38]. The function of any of these enzymes in cartilage is unknown.

*MMP3 *(stromelysin 1) is one of the most highly expressed proteases in cartilage and was significantly decreased in expression in OA in the current study and in our two previous studies of gene expression in cartilage [7,11]. The function of *MMP3 *in cartilage homeostasis is not certain, although it is capable of degrading aggrecan and also of activating procollagenases. It is possible that *MMP3 *has a maintenance function in cartilage that is lost in end-stage OA.

Pappalysin-2 is also decreased in expression in OA cartilage. This enzyme has been shown to degrade IGF binding protein-5 and to some extent IGF binding protein-3 [39], and therefore has the potential to control IGF availability in cartilage. Transforming growth factor-beta-induced chondrocyte proliferation was recently shown to be mediated by *ADAM12*-mediated degradation of IGF binding protein-5 [40].

Htra1 is a serine protease that has previously been implicated in cartilage destruction during OA [41]. Htra3 has not been associated with OA, but in this study the gene is expressed at a similar level in cartilage as Htra1 and is increased in expression in OA with comparable fold change and significance.

The expression of a number of proprotein convertases is altered in OA cartilage compared with normal cartilage, with the *PCSK6 *gene (PACE4) most highly regulated. Proprotein convertases are responsible for the activation of a number of proMMPs and proADAMTSs, with PACE4 recently identified as the enzyme that activates aggrecanases in chondrocytes [42].

Four genes encoding complement factors are regulated in OA cartilage compared with normal, with CFI, C2 and DF increasing and CFB decreasing. In the complement pathway, C2 is part of the classical pathway and CFB and DF are part of the alternative pathway with CFI inhibiting C3b. CFI was also recently described as increasing in expression in the lesion site of an OA knee compared with macroscopically normal cartilage from the same knee [43]. Other relevant functions for complement factors in OA have also been described, with C1s recently identified as the protease in OA synovial fluid responsible for cleavage of IGF binding protein-5 [44]. DF, also known as adipsin, is used as a marker of adipose cells and may be a readout for the differentiation status of the chondrocytes in OA cartilage.

*PROCL *is also called 'regeneration-associated muscle protease' or RAMP, and is induced in regenerating skeletal muscle in mice, as well as being lower in muscle cell lines derived from Duchenne muscular dystrophy patients compared with a normal cell line [45]. This protease may therefore have a role in tissue regeneration, pertinent to cartilage in OA.

As already briefly discussed above, several members of the rhomboid family of intramembrane proteases are expressed in cartilage and altered in expression in OA. *RHBDF1 *has recently been implicated in signalling from the epidermal growth factor receptor [46] that is implicated in skeletal development, as well as in autophagy [47]. PARL, a mitochondrial rhomboid, is a regulator of apoptosis [48].

We have previously shown fibroblast activation protein alpha to be elevated in OA cartilage and by inflammatory stimuli in chondrocytes [49].

The gene encoded by Hsp90AB1 is a cytoplasmic heat shock protein, whilst that encoded by Hsp90B1 is located in the endoplasmic reticulum; both genes are highly expressed in cartilage and significantly increased in OA. Heat shock proteins act as molecular chaperones, and their induction may indicate a level of cell stress. Heat shock protein 90 has also been shown to mediate IGF-1 and IL-1β signalling in chondrocytes, and to contribute to the expression of the *MMP13 *gene [50].

## Conclusions

There are myriad possibilities for protease function in cartilage metabolism, which may alter in OA, but a number of these come to the fore in the results and discussion above: direct proteolysis of extracellular matrix proteins or proteoglycans; activation of other proteases; regulation of cell signalling (for example, via IGF or IL-1); apoptosis and/or autophagy; and, related to this, intracellular degradation of proteins.

The present study is the most complete quantitative analysis of protease gene expression in cartilage to date. The data help give direction to future research on the specific function(s) of individual proteases or protease families in normal cartilage and in OA.

## Abbreviations

ADAM: a disintegrin and metalloproteinase domain; ADAMTS: a disintegrin and metalloproteinase domain with thrombospondin motifs; Ct: threshold cycle; IGF: insulin-like growth factor; IL: interleukin; MMP: matrix metalloproteinase; NOF: neck of femur; OA: osteoarthritis; PBS: phosphate-buffered saline; PCR: polymerase chain reaction; RAMP: regeneration-associated muscle protease; RT: reverse transcriptase; SUMO: small ubiquitin-like modifier; TNF: tumour necrosis factor.

## Competing interests

The authors declare that they have no competing interests.

## Authors' contributions

TES helped to design and coordinate the study, collected and processed tissue samples, performed real-time PCR, analysed data and helped draft the manuscript. JGW helped in collecting and processing tissue samples. RKD helped in collecting and processing tissue samples, and advised running and interpreting low-density arrays. CJP and XSP designed the low-density arrays. CD and AC took patient consent and coordinated tissue collection. STD helped design and coordinate the study, tissue collection and interpretation of data. GRG and WW undertook data analysis, particularly with respect to machine learning algorithms. IMC helped conceive, design and coordinate the study, analysed data and helped to draft the manuscript. All authors read and approved the final manuscript.

## Supplementary Material

Additional data file 1 Word file containing a table that lists full details of the LogitBoost-NR algorithms for gene selection and sample classification from gene expression data. Click here for file

Additional data file 2 Excel file containing a spreadsheet of expression data for all protease genes assayed (median threshold cycle (Ct) of NOF and OA, fold difference and *P *values). Click here for file

Additional data file 3 Excel file containing a spreadsheet of the rank order for all protease genes assayed by the LogitBoost-NR algorithm and the Wilcoxon test. Click here for file
