# Supplementary material for: Degradome expression profiling in human articular cartilage
Source: Arthritis Res Ther. 2009 Jun 23;11(3):R96. doi: 10.1186/ar2741 (PMC2714152; doi:10.1186/ar2741)
Supplement: Additional data file 1 — Word file containing a table that lists full details of the LogitBoost-NR algorithms for gene selection and sample classification from gene expression data. [file ar2741-S1.doc]

**LogitBoost-NR Algorithms**

**for gene selection and sample classification from gene expression data.**

LogitBoost-NR is a machine learning ensemble algorithm that was specifically developed by Guile and Wang [10, 53] in the School of Computing Sciences, University of East Anglia, to induce a series of decision tree classifiers using gene expression data for classifying samples, and then was extended for feature selection, i.e. identifying the genes most relevant to disease pathology. This supplementary document provides some details of these algorithms, specifically (1) explaining the general principles behind the boosting procedure for sample classification and presenting the LogitBoost-NR algorithm, and (2) describing how LogitBoost-NR is used for feature selection and giving the feature election algorithm.

**1. The LogitBoost-NR algorithm for sample classification.**

The original LogitBoost is a boosting algorithm for predictive classification [9]. It was first applied to gene expression data by Dettling and Bühlmann who used it with DNA microarray data [51]. They found that it gave lower error rates than the commonly used AdaBoost algorithm and concluded that this was because LogitBoost is less sensitive to outliers [51].

Boosting algorithms such as LogitBoost and AdaBoost are ensemble learning methods. The principle behind ensemble learning is that although a classification algorithm may only be able to produce a model with slightly better accuracy than random guessing, if several such models are produced and combined into an ensemble, their combined accuracy will be greater than any single classifier, providing they are sufficiently diverse from each other to avoid making similar errors. Boosting algorithms iteratively employ another algorithm known as the base learner to generate a series of models. Any algorithm normally used for classification or prediction can be employed as the base learner, providing it allows weighting of samples. Normally simple base learners such as decision trees are used and for gene expression data where the number of samples is usually small, simple decision stumps (i.e. decision trees with only one node) have proven very effective. Initially all samples have equal weights, then after the first iteration the accuracy of the model produced is measured and the samples weights are adjusted so that the weights of misclassified samples are increased while those of correctly classified samples are reduced. At the next iteration the base learner will concentrate on the misclassified samples. A series of models is then produced with the sample weights being adjusted each time. These models are then combined into an ensemble voting committee. Different boosting algorithms vary in the way that the sample weights are adjusted and the way the votes of the committee members are set. These differences may result in different overall performance on a given dataset, as Dettling and Bühlmann found when comparing AdaBoost and LogitBoost which differ in their weighting mechanisms. They reasoned that the lower error rates obtained with LogitBoost were because that unlike AdaBoost, which uses an exponential function, LogitBoost uses the binomial log likelihood, which increases linearly rather than exponentially for strong negative margins. Because of this, LogitBoost is more robust than AdaBoost when data are noisy or samples are mislabelled.

However, although LogitBoost can give better classification accuracy than AdaBoost, both of these algorithms were originally designed for datasets where the number of samples is much larger than the number of features, and this is not normally the case with gene expression data, where the number of features (genes) is usually much larger than the number of samples. Long and Vega [52] pointed out that with standard boosting algorithms if one feature correctly identifies all samples in the training data, that feature will continue to be selected for the remainder of the boosting process. Thus, other features will be ignored. This means that complementary information is not taken into account when building the remainder of the ensemble. One way to prevent this happening would be to apply feature non-replacement. This method removes a feature from the data once it has been used in a model, thereby forcing the base learner to only consider other data. This means that information complementary to the removed features must be taken into account when building further models. Long and Vega incorporated feature non-replacement into AdaBoost and found that their modified algorithm gave lower error rates than standard AdaBoost [52].

Because LogitBoost gives lower error rates than AdaBoost, we incorporated feature non-replacement into LogitBoost in a previous study and found that this improved its classification accuracy on microarray data [53]. Overall, LogitBoost-NR outperformed both standard LogitBoost and AdaBoost-NR. The algorithm for LogitBoost-NR is given below.

LogitBoost-NR with decision stumps.

1. Starting with *Ν* training pairs (**x**_1_,*y*_1_),…,(**x***_N_*,*y_Ν_*) with equal weights *w_i_* = 1/*N*, *i* = 1,…,*N*, function *F*(*x*) = 0 and sample probability estimates *p*(**x***_i_*) = 0.5.
   Let *E* be an ensemble of decision stumps, initially empty.
2. Repeat for *m* = 1,…,*M*:
   1. Compute the working response and sample weights:
      *z_i_* = [*y_i_* – *p*(**x***_i_*) ] / [*p*(**x***_i_*)(1 – *p*(**x***_i_*))],
      *w_i_* = *p*(**x***_i_*)(1 – *p*(**x***_i_*)).
   2. Fit the decision stump *f_m_*(**x**) by weighted least squares on **x** using weights *w_i_*.
   3. Update *F*(**x**) and *p*(**x**):
      *F*(**x**) ← *F*(**x**) + 0.5*f_m_*(**x**),
      *p*(**x**) ← (1+*e*^–2^*^F^*(**^x^**))^–1^
   4. Remove feature used in *f_m_*(**x**) from the training data.
   5. Add *f_m_*(**x**) to *E*.
3. Output *E*.

**2. Using LogitBoost-NR for feature selection**

A number of basic statistical methods have been applied to gene expression data for selecting the most relevant genes, including Student’s t-test, signal to noise ratio and the Wilcoxon-Mann-Whitney U-test (Wilcoxon test). These all allow genes to be ranked according to how well they discriminate between sample classes and thereby identify those most relevant to the pathology of a condition. However, these basic statistical methods perform feature selection by treating all samples equally and, in practice, some samples are more difficult to classify than others. Because boosting techniques are able to improve classification for these more difficult samples, and have been successfully used for the classification of gene expression data, an obvious further application is to use boosting for gene selection, and we have previously described an application of boosting to gene selection for microarray data [10].

Our boosting method for feature ranking applies LogitBoost-NR to a series of random partitions of the training data. The boosting algorithm produces an ensemble using each partition of the data and the genes are scored by the number of ensembles in which they are used. The genes can then be ranked according their scores. The details of the method are as follows.

A training dataset (e.g consisting of two thirds of the data) is randomly partitioned from a complete dataset. The LogitBoostNR algorithm is then applied for *M* iterations of boosting. Because decision stumps are used as the base classifier, when the non-replacement strategy is applied each iteration will generate one classifier that only uses one gene. Thus *M* different genes will be selected after *M* iterations to build an ensemble.

The process is repeated for *P* different random partitions of the data and the genes are scored according to the frequency of the number of ensembles in which they have been used. The algorithm for the feature selection process is given below.

Feature ranking algorithm using LogitBoost-NR.

1. Given a dataset *D* consisting of *Α* features and *S* samples, which can be represented by {(**x**_1_,*y*_1_),…,(**x***_S_*,*y_S_* )}, where **x** is a vector of the values of features *g*_1_,...,*g_Α_* (this is the expression profile of the genes) and *y* ∈ {0,1} is the class label for each sample.
   1. Let **r** be a vector of length *Α*, to contain the ranking scores **r**_1_,...,**r***_Α_* for features *g*_1_,...,*g_Α_*.
   2. Initialize **r** = [0,...,0].
2. Repeat for *i*= 1,...,*Ρ*:
   1. From *D* randomly select (without replacement) datasubset *L_i_* containing *Ν* samples where *Ν* is specified by the user.
   2. Perform *Μ* rounds of boosting on *L_i_*, using LogitBoost-NR, with decision stumps as base learner, to give ensemble *Ε_i_*.
   3. Increment the score in **r** by 1 for each gene used in *Ε_i_*.
3. Output **r**, the ranking scores of the features.

In our original application of this feature ranking algorithm we set *P*=50, *N*=2/3 of total samples and M=25 because these values gave satisfactory results for predictive classification using LogitBoost-NR. For the quantitative PCR data used in this study, which had smaller sample numbers than the DNA microarray data we used previously, we set *N*=1/2 of the total number of samples.
